# Enhancing Social and Emotional Learning: Insights from Student Teacher Reflections in the UAE and Finland

**DOI:** 10.3390/bs16010088

**Published:** 2026-01-08

**Authors:** Markus Talvio, Joona Vuorinen

**Affiliations:** Faculty of Educational Sciences, University of Helsinki, Siltavuorenpenger 5, P.O. Box 9, FI-00014 Helsinki, Finland; joona.vuorinen@live.fi

**Keywords:** social and emotional learning (SEL), social interaction skills, qualitative study, teacher training, reflection, learning journal

## Abstract

Numerous studies have demonstrated the benefits of social and emotional learning (SEL) for both students and teachers. For students, SEL enhances their learning environment, psychological well-being, and academic performance. For teachers, it fosters a sense of meaningfulness in their work and strengthens relationships with students. However, effective SEL requires guidance, a safe emotional environment, and opportunities for personal reflection. This study explored SEL by analyzing the reflections of eight trainee teachers in the United Arab Emirates and six in Finland, who participated in similar SEL courses independently. Utilizing both data-driven and theory-driven content analysis based on the levels of processing the results indicated that over 60% of student responses were elaborative or insightful, while less than 40% were at the reproduction level. The most prominent SEL category identified was developing social interaction, whereas responsible decision-making was the least represented. Overall, the course significantly enhanced participants’ theoretical understanding and SEL development, yielding similar outcomes in both countries. The findings suggest that methods promoting responsible decision-making should be further integrated into teacher training for SEL development.

## 1. Introduction

Extensive research consistently confirms the profound connection between social and emotional learning (SEL) and students’ overall school experience and academic success (Mahoney et al., 2018; J. Durlak et al., 2022). Comprehensive meta-analyses demonstrate that universal SEL programs significantly enhance the school atmosphere, improve feelings of safety, and improve student well-being, attitudes, behavior, and academic performance (Taylor et al., 2017; J. A. Durlak et al., 2011). This justification for prioritizing SEL is strengthened by recent global findings, including the 2024 OECD report, “The Survey on Social and Emotional Skills (SSES)” (Organisation for Economic Co-Operation and Development, 2024), and a systematic analysis conducted by Ha et al. (2025) involving over 33,000 students, all of which underscore the necessity of SEL skills such as empathy, emotion regulation, and resilience.

However, SEL skills do not develop on their own. Teachers play a central role, not only in teaching these skills but also in serving as role models of skilled social and emotional behavior and promoting a pro-social classroom environment. Indeed, high-quality education fundamentally depends on the social and emotional climate that teachers actively cultivate through their own skilled use of these competencies within their classrooms (Pianta et al., 2012). They are not merely facilitators of knowledge; rather, they are the essential architects of a supportive learning environment where all the elements of SEL can thrive (J. A. Durlak et al., 2011; Domitrovich et al., 2017; Talvio, 2014).

This research employs a qualitative methodology to explore the change process. Specifically, this study investigates the development and trajectory of trainee teachers’ professional thinking during SEL training in two countries.

### 1.1. Developing SEL: Core Competencies and the Role of Social Interaction Skills

Social and emotional learning (SEL), first introduced in 1994, is a conceptual framework with several core components, namely self-awareness, self-management, social awareness, relationship skills, and responsible decision-making (Elias et al., 1997). These elements enable individuals to identify their strengths and limitations and to develop positive feelings about themselves and others.

The benefits of SEL can be approached through intrapersonal and interpersonal competencies, which Gardner (1993) defined as essential for understanding oneself and social interaction (Lemerise & Arsenio, 2000). Developing self-awareness and self-management improves learners’ intrapersonal competence, allowing them to regulate their inner reality—emotions, needs, and wants—to reach goals. Interpersonal competence, which includes social awareness and relationship skills, is crucial for meaningful relationships and effective interaction. The third element, cognitive competence, advances collaboration and promotes responsible decision-making and ethical choices (Jones & Kahn, 2017) 

All of these competencies, including SEL elements, can be developed. Lintunen and Gould (2014) introduced a theoretical framework utilizing specific social interaction skills identified by Gordon (Gordon, 2019; Gordon & Burch, 2003) that enhances student learning of the core components of SEL. These skills include I-messages, active listening, the both-win method, and the skill of avoiding roadblocks (Gordon, 2019; Gordon & Burch, 2003). I-messages, in particular, foster self-awareness and self-management by enabling individuals to articulate their feelings and needs simply and truthfully, grounded in their personal perspective (Gordon, 2019; Gordon & Burch, 2003). To develop social awareness and relationship skills, active listening is an essential technique that involves the listener reflecting back their understanding to the speaker (Ivey et al., 2009). The both-win method, introduced to foster responsible decision-making, encourages respectful, impartial decisions by investigating the needs of all parties before selecting a solution (Adams et al., 2006; Gordon, 2019).

The Lintunen and Gould (2014) model provides tools for teaching social interaction skills, whose trackable learning outcomes enable and enhance the core components of SEL. Furthermore, the model’s inherent structure means that these skills also offer practical evaluation tools for assessing SEL’s components. Exploring the effectiveness of such tools is paramount, as teachers require dedicated support to ensure the successful implementation of SEL in the classroom, in addition to fostering their own SEL skills (Jennings & Greenberg, 2009; National Commission on Social, Emotional and Academic Development, 2018; Talvio & Lonka, 2021).

### 1.2. Prior Research on Teacher SEL Training and Its Outcomes

Ensuring that teachers are skilled in SEL is essential for effective teaching, with strong SEL skills directly supporting teacher well-being, enhancing their sense of meaning in their work, and improving interactions with students (Bottiani et al., 2019; Cipriano et al., 2023; Collie, 2017; Schonert-Reichl, 2017; Talvio et al., 2015). This link extends to the classroom, where strong teacher SEL skills have a well-established positive relationship with the classroom climate, student behavior, and academic outcomes (Jennings & Greenberg, 2009). SEL skills are known to be malleable—they can be learned and improved in adulthood but require maintenance to prevent decline (Organisation for Economic Co-Operation and Development, 2024; Talvio, 2014; Talvio et al., 2021).

Recognizing this need, research has explored the impact of SEL training for educators. Prior studies in Finland indicated that even highly educated teachers require support for developing their SEL skills. Evidence suggests that this training is immediately beneficial: even short, targeted interventions assessed through knowledge tests and scenario-based application showed significant improvements (Talvio, 2014). This research was then expanded through quantitative and longitudinal studies conducted across multiple countries. These international findings echoed the initial Finnish results, confirming a similar need among diverse educators and examining the sustainability of acquired SEL skills and teachers’ sense of competence (Talvio, 2014; Talvio & Lonka, 2019, 2021). Qualitatively, post-training reports indicated beneficial changes, with teachers using I-messages more effectively and creating environments that supported students’ autonomy and agency through better listening and problem-solving (Talvio et al., 2015).

However, while several studies indicate the immediate benefits of SEL training for teachers, we have scant information regarding how reflecting on SEL and related social interaction skills can support teachers and pre-service teachers in deepening their understanding of these concepts during their professional development. In this study, we will investigate trainee teachers’ reflections and levels of thinking while developing SEL skills.

### 1.3. Reflective Writing as a Tool for Deeper Understanding

The importance of reflective thinking for learning has been studied for a long time. Large meta-analyses show that reflective interventions in higher education, particularly reflective writing such as the use of learning journals, significantly improve the academic performance, critical thinking, cultural understanding, and metacognitive skills of university students (Guo, 2021; Sudirman et al., 2024).

The use of reflection journals has been linked to improving students’ self-reflection, learning motivation, and critical thinking skills (Alt et al., 2022; Brooman & Darwent, 2012; Essa, 2024). Regular use of reflection journals promotes stronger lifelong learning capabilities, including the ability for independent learning and self-regulation (Alt et al., 2022; Wallin & Adawi, 2017). Furthermore, learning journals help first-year students to adapt to the university environment and develop required academic study skills (Brooman & Darwent, 2012). This method is also considered effective for critically processing previously learned material and deepening current understanding (Lew & Schmidt, 2011; Brooman & Darwent, 2012).

Challenges in implementation, however, persist. Lew and Schmidt (2011) found that while 3460 first-year students generally considered journal writing to be useful, the benefits were not uniform. Many students, for instance, required extensive support and clear instructions to structure their reflective writing effectively. Furthermore, accessing the full benefits of learning journals often requires sufficient time and clear feedback, extending beyond the assignment itself (Essa, 2024).

Therefore, to ensure a smooth learning experience, students’ familiarity and skill level with reflective writing must be taken into account when integrating learning journals into training. Similarly, clear instructional guidelines for teachers are crucial for multiple reasons: they allow teachers to effectively guide students toward effective reflective practice, and they enable educators to better evaluate the quality of student reflective texts and, consequently, their learning level.

### 1.4. Levels of Processing in Reflective Writing

The theoretical foundation of this study is based on the unpublished three-level process classification developed by Talvio and Lonka (2025), which is rooted in the two original categories established by Lonka and Mikkonen (1989). They initially created their categories to distinguish between surface- and deep-level learning in students’ essay responses during a national examination. The units representing surface learning were categorized as reproduction. In these answers, information was presented as depicted in the source without deeper reflection, akin to the concept of Knowledge Telling (Bereiter & Scardamalia, 1987; Scardamalia & Bereiter, 1991). The second category, elaboration, reflected deeper learning, where the writer provided a rationale or drew conclusions based on facts. This corresponds to Knowledge Transforming and requires the writer’s conscious effort and engagement (Bereiter & Scardamalia, 1987; Scardamalia & Bereiter, 1991). Deep processing (elaboration) has been linked in subsequent research to attaining a broader and better understanding of learned information, increased learning commitment, and more long-term, applicable learning (Lonka et al., 1994).

However, while analyzing trainee teachers’ learning journal summaries, Talvio and Lonka (2025) found that some students surpassed these initial two levels by critically reflecting on their prior thinking and articulating new insights or shifts in their understanding. Consequently, they introduced a third category to the classification: critical self-reflection. This category represents the highest level of learning and reflection—a shift in thinking or gaining a new insight—and is comparable to the highest levels found in many hierarchical reflection models (e.g., Van Manen, 1991; Hatton & Smith, 1995).

The three-level process classification (Talvio & Lonka, 2025) was first used to investigate trainee teachers’ learning during their introduction course on educational psychology. Given the classification’s general nature and broad applicability, a tailored version for the evaluation of SEL and its components was employed.

### 1.5. Aim of This Study

This study analyzes responses using the three hierarchical processing levels defined by Talvio and Lonka (2025): reproduction, elaboration, and critical self-reflection. The hierarchy signifies increasingly deeper reflection on and more extensive description of learning as one moves through the levels. By analyzing reflections collected during trainee teacher SEL training, this study explores the process of social and emotional learning. Specifically, we seek to understand how participants process SEL issues, how they might consolidate learning for real-life application, and how they might develop novel insights. Accordingly, the following research questions are addressed:

During the course on social and emotional learning (SEL):

- At which level of processing (Talvio & Lonka, 2025) do trainee teachers reflect on their learning?
- What core competencies and skills related to SEL do trainee teachers reflect on and how they are being processed?
- Are there any country-specific findings in trainee teachers’ responses?

We are also interested in examining whether the general three-level process classification (Talvio & Lonka, 2025) can effectively distinguish between different levels of SEL, and whether such a general classification can also be applied to its evaluation.

## 2. Materials and Methods

### 2.1. Context

The objective of the teacher education in *Finland* is to train autonomous, reflective, and investigative teachers through utilizing diverse methods in pedagogy (Toom et al., 2010; Tryggvason, 2009). Teachers are encouraged to make pedagogical decisions based on a solid theoretical foundation and engage in reflective practice as professionals (Talvio, 2014). Additionally, it is crucial for student teachers to gain practical experience in the field. Five-year teacher education at all Finnish universities is research-based and has been taught at MA level since 1974. The organization of activities within teacher training encourages students to engage in argumentation, decision-making, and justification, as well as the exploration and resolution of pedagogical challenges.

Teachers in Finland also have an administrative-level role and collaboratively adapt the national core curriculum to the specific needs of their own school and individual classrooms. This allows teachers to create a customized school curriculum that takes into account local circumstances (Toom et al., 2010). This internal localization is enabled by the comprehensive university training that emphasizes the development of teachers as autonomous pedagogical experts through research-based teaching, reflection, critical thinking, and collaborative skills (Lonka, 2018). Consequently, student teachers in Finland are also equipped to adapt and tailor the curriculum to meet the diverse needs of their students in inclusive classrooms.

In the *United Arab Emirates (UAE)*, teachers must have a minimum of a bachelor’s degree or a 4-year university degree or higher in the required field. This rule applies for public/government schools, as well as private schools, in the UAE. Education policymakers have actively worked on crafting curricula that incorporate targeted learning objectives in line with global standards, alongside implementing a robust teacher licensing system (Hathorn & Dillon, 2018). Recently, numerous reforms have focused on the professional development of teachers, which is emerging as a crucial element of education reform efforts. Overall, one way of improving the quality of teaching and learning globally is promoting collaboration between teacher training institutions in different countries (Talvio et al., 2023).

In the UAE in particular, international collaboration in higher education has a long history (Mahani & Molki, 2011). The goal of the present collaborative project in education between institutions in the UAE and Finland was to develop trainee teachers’ professional competencies by ensuring that they receive high-quality teaching knowledge and skills from educators from around the world (Lonka, 2018). This study investigates the development of trainee teachers who participated in SEL courses created through collaboration between the UAE and Finland.

### 2.2. Content of the Courses in the UAE and Finland

The social and emotional learning (SEL) course conducted at the Sharjah Education Academy (SEA) in the United Arab Emirates was developed from the Group Process and Interaction in Educational Psychology course offered to student teachers as their first bachelor-level course at the University of Helsinki (UoH), Finland. The goal of both courses is to promote group processes, enhance interaction in various situations, and acquire and apply the theory of SEL and social interaction skills (Gordon, 2019; Gordon & Burch, 2003). The learning outcomes of the course were culturally adapted to meet the national requirements of the UAE. However, the literature studied in these two courses was similar and the same teacher conducted both courses. The length of both SEL courses in the UAE and the Group Process and Interaction in Educational Psychology course at the UoH was 5 ETCS modules organized in the first semester. Assessment of students’ progress was carried out mostly in a formative way. The study material consisted of readings and videos; students studied individually and in small groups. The assignments included, for example, reflection papers, simulations, and small group presentations in both courses. These shared goals, structure, and teaching methods indicate that the two courses were fundamentally similar, providing consistent learning experiences despite being implemented in different cultural contexts.

### 2.3. Participants

The cohort from the UAE participating in the SEL course consisted of eight students enrolled in a one-year Post-Graduate Diploma in Education (PGDE) program at the SEA. The participants, who were in-service teachers, represented several nationalities. Additionally, six pre-service student teachers from the UoH who participated in the Group Process and Interaction in Educational Psychology course took part in this study. All of them were originally from Finland. Although the two groups had different levels of work experience, their prior pedagogical studies were limited before this program.

### 2.4. Materials

The data used in this study consisted of one of the course assignments: a summary of the students’ learning diaries. Throughout the entire duration of the course, students were expected to reflect on their learning experiences, which was intended to support and deepen their understanding of the course content. Participants were encouraged to freely express their thoughts on the course, including their expectations, aspects that they found interesting or important, and any disagreements or challenges that they encountered. At the end of the course, students were asked to summarize their learning diary entries by highlighting the most significant content for evaluation. These summaries, which ranged from 2 to 3 pages in length, served as the basis for this study.

### 2.5. Analysis

This study employed a phenomenographically inspired approach that emphasized the classification of ideas rather than the students who expressed them (Marton & Booth, 1997). First, researchers carefully read through each answer multiple times to pinpoint segments of text that informed aspects of the research question. Only reflections that explicitly addressed what SEL skills and knowledge the students learned during the course were included in the analysis. A single notion, view, or opinion expressed by a participant was treated as one unit of analysis. While 247 units were initially identified, only 209 fell within the scope of this study (122 from the SEA students’ summaries and 87 from the UoH students’ reflections).

Two content analyses were conducted to analyze the data. The first analysis was conducted to investigate the themes of students’ reflections. Building on the SEL theory developed by Elias et al. (1997), the analysis initially used its five core elements for categorization. The framework was then adapted to more clearly organize the data. Self-awareness and self-management were merged into a single category, and likewise, social awareness and relationship skills were combined. Given the strong emphasis on group dynamics in both SEL courses at the UoH and SEA, a new, separate category for group dynamics was established from the original relationship skills category. The final categories for the analysis were developing social interaction, group dynamics, self-regulation, and responsible decision-making.

Drawing on the levels of processing categorization, a second content analysis explored students’ learning levels (Talvio & Lonka, 2025). It included three levels of processing: reproduction, elaboration, and critical self-reflection. In *reproduction* units, information was presented exactly as it appeared in the source. The *elaboration* category, on the other hand, involved the writer providing reasons for or drawing conclusions based on the facts. The highest level of processing was *critical self-reflection*, in which previous thoughts were critically examined and new insights or changes in understanding were articulated (Talvio & Lonka, 2025).

During the categorization process, the researchers employed peer debriefing (Miles & Huberman, 1994) to achieve consensual validation. Recognizing that the absence of specialized software could pose risks to systematicity and transparency, we implemented a rigorous quality assurance protocol to mitigate these concerns. Expert peer debriefing was central to this protocol, ensuring reliability and validity throughout the analysis. The co-author, a specialist in applying the structured three-level processing framework developed by Talvio and Lonka (2025), provided continuous oversight and guidance. Through expert debriefing, emerging themes and classifications were critically reviewed, challenged, and confirmed, ensuring strict adherence to the methodological framework. Furthermore, the research team engaged in ongoing discussions to evaluate and validate specific unit classifications. These collaborative deliberations reinforced systematicity and methodological rigor, ensuring the robustness of the analytical process.

Theoretical saturation of the themes was reached during the analytical phase of the study. While data collection did not strictly adhere to the saturation principle due to the use of pre-existing data, the inductive analysis was finalized once specific criteria were met: no new categories emerged, as the constant comparative method revealed no additional core concepts, themes, or relationships necessitating modifications to the established category structure; and the final categories demonstrated conceptual density, supported by diverse illustrative examples that ensured a comprehensive exploration of the phenomenon.

The completeness of the analysis was further reinforced by the robustness of the data source. The dataset, comprising 209 learning diary summaries, provided sufficient breadth and depth to capture variations in participants’ reflections. Additionally, the systematic application of Talvio and Lonka’s (2025) framework facilitated an iterative and rigorous examination of the dataset, confirming the achievement of theoretical saturation.

### 2.6. Ethical Considerations

This study was conducted in accordance with strict ethical guidelines. All participants received a detailed explanation of the data collection process and privacy measures used. We guaranteed that all data and responses would remain anonymous and confidential, and we invited students to participate only after their final course grades were released to prevent any impact on their academic standing. Participants were explicitly informed of their right to withdraw from this study at any time without consequence. This study’s purpose was explained, and participant anonymity was maintained by removing all identifying information. None of the participants asked for their learning journals to be removed from the data. Researchers conducted the analyses themselves to enhance trustworthiness and ethical consideration.

To protect the confidentiality of the student teachers at the SEA in the UAE, their nationalities were anonymized. As these were the first courses of their kind in the Middle East, this step was crucial to ensure that no individual could be identified. To further protect their anonymity, the results section does not specify the countries of origin of the students or the country in which the study took place.

## 3. Results

The results of this study are structured thematically around the four SEL categories. Each SEL category is then subcategorized and analyzed according to its level of processing (Talvio & Lonka, 2025). Categories are arranged by size (developing social interaction, group dynamics, self-regulation, responsible decision-making), and the subcategories within each category follow this sequence: reproduction > elaboration > critical self-reflection. Table 1 details the complete numeric and percentage breakdowns of all categories and subcategories for each country. After outlining the major, shared results of both countries, the textual analysis concludes with a short observation on select country-specific findings. This structure reflects this study’s primary aim of concentrating on similarities, rather than differences, between the two countries.

### 3.1. Developing Social Interaction

The biggest category, covering almost 46% of all SEL categories, was developing social interaction, which had 97 analysis units. Altogether, 23 responses (24%) represented *critical self-reflection*. Active listening (Gordon, 2019; Gordon & Burch, 2003) was considered an important skill for expressing one’s needs while also showing empathy and understanding. It was viewed as a way to be genuinely present in conversation with another person, focusing on connection rather than on a quick fix. This skill demonstrates the foundational human need to understand and help others, which is crucial for dealing with prejudices and cultural differences.

I now realise that socially and emotionally competent teacher possess high social awareness. Social awareness for teachers makes them to think from the other person’s perspective i.e., to keep aside the biases and put yourself on the shoe of another person. Same way, students will also think from other’s perspective. I witness my students being empathetic since they have a wheelchair mate with whom, my students show love, care and acceptance. This proves that we have to have a willingness to learn and the skill to listen to other people.

… If you really listen to someone, they feel valued and important thereby you become empathetic. It was significant to learn that active listening is a skill that the listener is reflecting back to the speaker’s words. Careful listening provides us the opportunity to get familiar with cultural differences as well.

Students’ responses revealed insights about recognizing and avoiding “roadblocks” (Gordon, 2019; Gordon & Burch, 2003), For instance, they found that avoiding name-calling and labeling was a good tool for improving the atmosphere in the classroom.

Previously, I used to have a lot of problems keeping my students in their groups particularly at the storming stage.—I would waste precious minutes of the lesson, trying to solve disputes. Once I acquired SEL skills, I figured that their frequent use of labels and roadblocks was a limiting factor. Some of them did not realize that it was not okay to label their class mate ‘lazy’ ‘slow’ ‘copycat’ and so we had to work on it together.—the focus shifted from label to fixing behavior and there are now improvements.

The use of I-messages (Gordon, 2019; Gordon & Burch, 2003) was perceived to be crucial for giving positive feedback and discussing things, particularly with children whose behavior frequently requires attention or correction.

I’ve started using I-messages to praise kids at work. I’m trying to find the most natural and respectful way to talk to children and using genuine praise works well. With this method, I explain what they did and how it made me feel. I think this is especially important for children who often get corrected for their behavior. I’ve made an effort to focus on even the smallest positive actions, especially with them.

Altogether, 38 out of 98 responses (39%) were categorized into the *elaboration* category. For example, they included examples of how the topics of the course can be used in the classroom or more generally in interactions between two people: “Using active listening and verbalizing another person’s feelings from between the lines can help them, for example a child, understand what they are feeling, what the feeling stems from, and what they can do about it.” (HL1). “Instead of adding to the problem, active listening gives the responsibility to the other person and allow them to come up with their own solutions.”

Out of 98 responses, 37 (38%) were in the *reproduction* category (Table 1). This is almost the same number as those found in the elaboration sub-category of the developing social interaction category. The themes of the course were handled at a superficial level, for example, by referring to the course literature or by repeating information provided in the course: “As Talvio and Klemola (2024) state, active listening and other factors for effective social interaction (such as I-messages) require active practice.”

### 3.2. Group Dynamics

The second biggest SEL category was group dynamics, covering 53 units, or 25%, of all 209 SEL responses. The group dynamics category included themes on how to formulate a group, the phases of the group, and the roles of the group. There were 11 units in the *critical self-reflection* category, indicating that 21% of the responses were at the highest processing level for this category. The importance of team building emerged from both the study group’s experiences and the perspective of their future roles as teachers.

It has been eye-opening to simultaneously be in the middle of the whirlwind of being a team-building participant, to observe the reasons for the course instructor’s investment in team building, and finally, to experience the effects of the team-building activities. The experience has gone so deep that I don’t believe it will be forgotten when it’s time to take on my own student group. At that time, I will remember how carefully they should be introduced to one another.

The answers included reflections on the group assignments and how they affected the group. Teambuilding was also mentioned as an important element of a functional group.

Through the exercises and self-directed group meetings, it became clear just how enriching a learning tool a functional group truly is. I noticed that I eventually approached the exercises—which I had previously known as ‘get-to-know-you games’—with much more openness, willingness, and enthusiasm than I would have expected.

Activities such as sharing group roles and breaking into smaller groups were seen as significant. They helped participants to join in and learn valuable new skills as teachers.

I once had to be a presenter on the basis that I was the one who wears the largest shoe size in my group. For me, it is an interesting and flexible way to promote interaction as everyone would get at least a chance to participate, even the shy ones. I have started using this method with my students and it has proved very useful in promoting a flourishing learner-centered classroom atmosphere.

Participants’ responses also reflected the phases of the group process. Critical reflections highlighted the importance of participants getting to know one another to create a safe and motivating environment.

Also, I always make sure not to rush the forming stage of our group creation process so students can get to know each other and bond really well. I believe that this phase is very important one in group dynamics. When students feel safe around their teammates, they will be motivated to work.

The largest subcategory within group dynamics was *elaboration*, accounting for 42% of the answers. Its 24 responses included examples of the group processes of the course and how the themes of the course are addressed in a school context: “My biggest takeaway from the group process was the importance of both a safe group atmosphere and an understanding of one’s personal responsibility within the group.”

At the *reproduction* level, the theme was reflected superficially, often through mere citation of course references or by repeating course content: “The group leader plays a very important role in moving beyond this superficiality (Talvio & Klemola, 2024).” Eighteen responses (34% of the units of analysis in this category) fell into this subcategory.

### 3.3. Self-Regulation

The self-regulation category included elements of self-awareness and self-management, key components of SEL. The responses reflected, for example, the ability to recognize one’s needs and goals, regulate one’s emotions, and use ‘I-messages’ and self-reflective explorations. Altogether, 43 units (21%) fell into this category.

Responses at the *critical self-reflection* level were quite typical, with its 15 answers covering almost 35% of this category. Self-awareness and self-management were seen as foundational for expressing oneself and interacting with others. This was reflected in the perspectives of students:

I believe that when one is aware of their own thoughts, emotions, strengths, limits and weaknesses, they will be able to express themselves and interact better with others. This self-awareness is a prerequisite to self-management, and both are essential SEL skills that students need to be trained on if we are to help them promote interaction and positive relationship building skills.

Some participants stated that they had been using social interaction skills unconsciously before the course without realizing it. Recognizing these skills helped them to further develop their abilities and use them consciously to improve their teaching:

This course made a big impact on me as a person, more as a teacher. When we began, I thought I didn’t have any knowledge on the topic, but I discovered that I already applied it in different contexts but without knowing what it really is. I wanted to learn more strategies to approach students, promote interactions and learn ways to make my classroom positive and friendly but also educating and successful.

The importance of self-awareness and self-management was highlighted by the psychological symptoms caused by the COVID-19 pandemic, particularly concerning reduced self-esteem. The value of reflection as a skill, along with support from one’s environment, was also emphasized:

During complex phenomenon such as COVID-19, pandemic, social interactions are different. Students might start developing negative feelings due to lack of support from their peers or the required support from teachers due to the restrictions and this might produce long lasting damage to students’ self-esteem.—Students could benefit by reflecting on their experiences and getting support. Therefore, self-awareness and self-management become extremely important as social and emotional learning competencies.

Analysis of the responses on self-management revealed key themes regarding understanding one’s basic needs. Setting and achieving goals were also emphasized:

At the beginning, I set a goal to stay motivated and engaged with the course and its content for the entire duration. Now, at the end, I’m satisfied with myself because I’ve reached that goal. The best part is that I’m probably even more interested in these topics than I was before the course started. Furthermore, my initial difficulties have lessened because I’m now more engaged with topics like developing social interaction skills and the processes of SEL.

*Elaboration*-level responses included practical examples of how the course content can be applied by a teacher and implemented in the classroom. Altogether, 17 responses (40%) fell into this subcategory. This example used the metaphor of an iceberg:

Getting angry is the peak of the iceberg, but beneath the surface are many other feelings that better explain the root cause and context of the anger we experience. This deeper understanding allows others to better comprehend and react to the situation.

At the *reproduction* level, the theme was handled superficially, for example, by citing the course literature and repeating the course content. This included direct quotes such as “The feelings that we express (secondary feelings) do not always explain what we really feel (primary feelings),” or explaining one’s development without giving examples that apply the concepts, such as “Now I feel that I have given explanations to my previous thinking and I have understood theories on thinking such as those related to self-awareness”. Altogether, 11 units of analysis (26%) categorized under self-regulation category fell into this reproduction subcategory.

### 3.4. Responsible Decision-Making

The smallest category was responsible decision-making, with 15 responses representing 7% of the units of analysis. Responses for *critical self-reflection* covered topics such as ethical issues, equality and consideration of others, and aspects of educational policy. Teachers’ responsibility and ability to act more ethically were emphasized as themes in this category. For example, group development and ensuring that nobody is excluded when the group is formed was reflected from an ethical perspective:

In my opinion, allowing the group members to form their own groups when they barely know each other doesn’t really serve anyone. The risk of ‘cliquing up’ would be too great. No one wants to make others feel excluded, nor does anyone want to be excluded themselves. I believe that, regardless of which side of the situation one is on, every person can relate to the uncomfortable feelings caused by loneliness, exclusion, or being left out. Furthermore, I feel it’s good to encourage people out of their comfort zones so they don’t ‘get stuck’ just with familiar, safe people. After all, by the time we reach working life, we face the reality that we must or must learn to get along with everyone. The richer the opinions and the more diverse the group, the more varied and interesting the perspectives that can emerge.

While labeling and roadblocks reveal a lack of personal self-management skills, an agreement made in the teacher lounge to avoid them can be considered an act of responsible decision-making:

As teachers, avoiding labels for students is quite significant in their overall well-being.—Very often teachers in the staff room tend to pass casual remarks about students which should be avoided and the underlying reason for student’s behavior should be focused.

Some answers highlighted phenomenon-based learning and emphasized the importance of creative and critical process of collaboration:

I can now enable my students with skills to carry out simple research, gather and evaluate data and present ideas.—Students will need to learn how to brainstorm ideas collaboratively and creatively and critically evaluate them for presentation.

*Elaboration*-level responses provided examples of the course content or demonstrated how specific content could be taught or used in the classroom. One respondent stated “Explaining the reasons behind my decisions is the core basis of respectful interaction, and I try to avoid relying on my authoritative role when creating roles or giving instructions.”

At the *reproduction* level, the course content was repeated or dealt with in a superficial manner: “Making responsible decisions and ethical choices are aspects of cognitive and social competence, including respectful and democratic methods when acting and working together.”

### 3.5. Some Country-Specific Findings

Table 1 shows that over half (55%) of the responses from the UAE were categorized into the developing social interaction category, whereas in Finland, only 37% fell into this category. Within this category, critical self-reflection accounted for a much larger proportion of the Finnish responses (41%) than the UAE responses (14%). Conversely, the remaining subcategories, reproduction and elaboration, were dominant in the UAE, with each accounting for over 40% of its responses. In Finland, these two subcategories were much smaller, each covering under 30% of the responses.

Another notable difference between the countries was identified in the group dynamics category, which accounted for 35% of Finnish responses but only 18% of UAE responses. Regarding subcategories, reproduction had similar weights in both countries, accounting for about one-third of the responses in this category. However, the other two subcategories showed a clearer divergence. In Finland, elaboration was the dominant subcategory, covering half (50%) of the group dynamics responses, while the critical self-reflection subcategory was smaller at 17%. Conversely, the UAE showed a more balanced distribution: elaboration accounted for 38% of responses in this category, and the percentage for critical self-reflection was larger than that of Finland at 29%.

In the responses of SEA students from the UAE, examples were often presented with reference to teachers’ work and responsibilities. This sense of teachership was often linked to the writer’s role by using the pronoun ‘we’ when referring to teachers. SEA students often considered social and emotional skills from the perspective of their pupils, not just as the teacher’s own skills. Since the participants from SEA generally had years of teaching experience, comparing their own teaching practice and providing examples related to their own pupils was understandable. Comparably fewer teacher-related responses were found from UoH students from Finland, presumably because, as first-year student teachers, they had less teaching experience, although individual differences existed. Furthermore, UoH students had likely not had time to develop their identities as teachers in the same way as SEA students. At the beginning of their studies, they may still perceive themselves more as ‘students’ than ‘teachers’, with this identity remaining until they gain more experience working as a teacher.

## 4. Discussion

This study analyzed the reflection levels and thematic content of trainee teachers’ learning journal summaries across two distinct cultural contexts following an SEL course. The main results indicated that the majority of responses demonstrated elaboration or self-reflection, showing that students were able to apply the studied content and gain new insights. This suggests that the course effectively stimulated the theoretical conceptualization of SEL and offered valuable insights for enhancing a supportive learning environment.

Analyzing thematic content revealed a differential learning trajectory across SEL components constituting another core finding. The most frequent category was developing social interaction. This analysis reveals the trainee teachers’ understanding that positive feedback operates as a strong antecedent for behavioral achievement. Crucially, their recognition of ‘road blocks’ indicates an awareness of the potential for teachers’ linguistic strategies to inadvertently deteriorate the classroom climate or otherwise negatively influence the quality of the teacher-student interaction. The salience of group dynamics confirms that participants perceive a positive classroom environment as essential for successful learning outcomes and understand the teacher’s decisive agency in its creation.

This prevalence is likely due to participants’ pre-existing professional knowledge structures (Schank, 1982). As all participants have prior experience with teaching, the observable aspects of interaction and learning atmosphere are highly familiar and readily articulated. Conversely, responsible decision-making emerged as the smallest category, comprising only 7% of the units of analysis. This finding suggests that while the concept was explicitly introduced during the course, its application remains less integrated into the trainee teachers’ developing professional knowledge structures than other ethical domains. The limited frequency of discussion on decision-making implies that the practical necessity of translating ethical awareness into actionable, deliberate choices may not yet be fully incorporated as an inherent and central aspect of their professional practice, despite their demonstrated overall awareness of teaching’s ethical dimensions. This is understandable, as responsible decision-making is often considered the most complex SEL component because it requires simultaneous integration and application of all SEL skills. For problem-solving and ethical decision-making, the literature emphasizes requirements such as the awareness and management of feelings and needs, including using I-messages (Gordon, 2019), as well as adaptive response strategies, including listening skills (Adams et al., 2006; Elias et al., 1997; Gordon, 2019). Consequently, the successful, holistic management of these SEL components necessitates deliberate practice, especially during the initial stages of skill acquisition. This difficulty leads to an uncommon focus on reflection on this complicated concept, as beginners often struggle to recognize these complex moments or successfully utilize their emerging skills and derive insights in real-life situations.

Regarding reflection depth, critical self-reflection was the smallest category in the UAE, consistent with prior research (Talvio & Lonka, 2025). In contrast, the smallest category for Finnish students was reproduction, suggesting a preference for critical evaluation and insight—a skill emphasized in Finnish pedagogy—over rote memorization. This difference may highlight how prior educational backgrounds influence the articulation and depth of reflective practice.

The unequal mastery of SEL components is a frequently observed phenomenon (Talvio et al., 2022) explained by both the implementation of training and developmental factors. From an implementation perspective, the weaker emergence of complex concepts such as responsible decision-making may stem from inconsistencies in program delivery, such as varying emphasis on components (Lonka & Talvio, 2021) or school standards that only partially address SEL (Schonert-Reichl et al., 2015). This reinforces the critical role of program fidelity in SEL training (J. A. Durlak, 2016). Furthermore, the results reflect the natural, non-linear progression of SEL. Conceptualized as a deepening spiral pathway, development begins with intrapersonal competence before progressing to interpersonal competence or cognitive competence (e.g., responsible decision-making). This sequential structure, mirroring Vygotsky’s internalization/externalization process (Vygotsky, 1930–1934/1978), suggests that the partial learning observed may be a natural artifact of SEL’s phased and hierarchical developmental structure, rather than an intervention failure.

As SEL is inherently culturally and contextually embedded (Humphrey, 2013), a critical trade-off exists between achieving high ecological validity and maintaining strict academic rigor. This inherent complexity dictates the qualitative methodology and imposes specific limits on the transferability of the findings. This study addresses this challenge by employing a qualitative approach that seeks to capture participants’ experiences and the cultural variations in the environments in which they live and work (Gegenfurtner et al., 2009). This research offers insights into the large variation in teacher learning, rather than focusing on comparisons between participants or their cultural or educational backgrounds. Specifically, this study investigates the development and trajectory of trainee teachers’ professional thinking during their SEL training.

Social desirability bias may have influenced students’ summaries of their learning diaries, leading students to write what they believe their instructor wanted to read. We believe, however, that deep, critical reflection is difficult without first achieving deep understanding. In fact, the dynamic might be the reverse: a student intending to please the instructor may remain at a superficial level, merely praising the teacher and the course content without genuinely elaborating or reflecting on it critically.

Due to the small sample size, readers should note that the results cannot be generalized. However, the aim of this qualitative study is not statistical generalizability, but rather to deepen the understanding of the SEL process and demonstrate the applicability of the general classification proposed by Talvio and Lonka (2025) to this area of research. The classification proved effective, successfully distinguishing three distinct levels of reflection among the trainee teachers and providing valuable insights into their developmental progression in SEL skills. In addition, while our study indicates that most participants could elaborate and critically reflect on SEL, this cognitive achievement does not guarantee that they will translate these skills into effective socially and emotionally skilled behavior in the classroom. However, a foundational understanding of the concept is necessary for its successful implementation in both personal and professional life. Therefore, we can conclude that deep reflection on SEL is a prerequisite for skilled social and emotional practice.

### Future Directions

The finding that partial learning is a natural artifact necessitates a key educational implication: future programs must consciously address the developmental lag of more recently emerging components such as responsible decision-making. Interventions should devote more time to program fidelity and, more critically, structure content to explicitly promote responsible decision-making by combining and applying previously studied SEL skills. This requires moving beyond defining skills based on challenging students to synthesize their self-awareness, social awareness, and relationship skills in complex, practical scenarios, thereby building the skills necessary for professional development and complete understanding of SEL. The scenarios gathered from trainee teachers offer a rich resource for developing instructional materials. These materials could include exercises designed to cultivate self-awareness, specifically through the identification of personal emotions and underlying needs. Furthermore, the scenarios are suitable for creating activities, such as role-playing, aimed at enhancing listening capacity and reinforcing social awareness. Incorporating the practice of problem-solving skills and responsible decision-making would ensure comprehensive coverage of the core competencies within SEL. A key pedagogical consideration, however, is the requirement for students to master the foundational social interaction skills individually prior to engaging in complex activities like problem-solving and responsible decision-making, which demand the synthesis of these skills.

For future research, several important directions emerge. Firstly, it would be valuable to investigate how pedagogically knowledgeable and competent participants reflect on their learning and synthesize the SEL course content with their existing theoretical understanding of pedagogy and educational psychology. Secondly, exploring the practical translation of learning outcomes is critical: specifically, it is important to understand how participants who achieve deep learning and critical self-reflection enact their SEL competencies through expert role-modeling and high-quality instruction, versus those who only reproduce content. Finally, to address the constraints on generalizability imposed by the small sample size, future studies should employ a larger and more diverse sample to validate these findings for broader application.

The findings of this study indicate a difference in the application of SEL based on professional experience: in-service teachers demonstrated a greater propensity to integrate SEL directly into the school context, whereas pre-service teachers, owing to their limited work experience, mainly reflected their learning through the lens of their personal lives. This observation prompts an important question regarding the transferability of their SEL competence. Consequently, further investigation is warranted to assess the sustainability of pre-service teachers’ SEL education and its capacity to be effectively utilized in their future professional practice as certified teachers.

Social and emotional learning (SEL) represents a critical dual investment, benefiting both students and educators who guide them. While the data confirm SEL’s power to enhance participant performance and well-being, its corresponding value lies in equipping teachers with essential SEL skills. In a rapidly evolving educational landscape, this competence is the bedrock upon which teachers can effectively and continuously adapt their practice, ultimately securing the future success of both the student and the system of schooling itself.

## Figures and Tables

**Table 1:** The number and percentage of units in the categories and subcategories for each country.

	Levels of Processing							
	Reproduction		Elaboration		Critical Self-Reflection		Total	
	*n*	%	*n*	%	*n*	%	*n*	%
*United Arab Emirates*								
Developing social interaction	27	42	28	44	9	14	64	55
Group dynamics	7	33	8	38	6	29	21	18
Self-regulation	7	26	11	41	9	33	27	23
Responsible decision-making	2	40	1	20	2	40	5	4
Total	43	37	48	41	26	22	117	100
*Finland*								
Developing social interaction	10	29	10	29	14	41	34	37
Group dynamics	11	34	16	50	5	16	32	35
Self-regulation	4	25	6	37	6	38	16	17
Responsible decision-making	0	0	6	60	4	40	10	11
Total	25	27	38	41	29	32	92	100

## Data Availability

The raw data supporting the conclusions of this article will be made available by the authors on request.

## Abbreviations

The following abbreviations are used in this manuscript:

ETCS	European Credit Transfer and Accumulation System
MA	Master of Arts
PGDE	Post-Graduate Diploma in Education
SEA	Sharjah Education Academy
SEL	Social and emotional learning
UAE	United Arab Emirates
UoH	University of Helsinki
